# Quantitative Analysis of Coronary Vessels with Optimized Intracoronary CT Number

**DOI:** 10.1371/journal.pone.0085312

**Published:** 2014-01-07

**Authors:** Sei Komatsu, Teruaki Kamata, Atsuko Imai, Tomoki Ohara, Kazuaki Miyaji, Yasuhiko Kobayashi, Kazuhisa Kodama

**Affiliations:** 1 Cardiovascular Center, Amagasaki Central Hospital, Hyogo, Japan; 2 Department of Diagnostic Imaging, Amagasaki Central Hospital, Hyogo, Japan; 3 Department of Cardiology, Osaka University School of Medicine, Osaka, Japan; Tokai University, Japan

## Abstract

**Background:**

Variability in intracoronary computed tomography (CT) number may influence vessel quantification. We confirmed the feasibility of a novel method for measuring vessel diameter and area using coronary CT angiography (CCTA) with an optimized intracoronary CT number, 350 HU.

**Methods:**

We performed intravascular ultrasound (IVUS) imaging in 52 patients with significant stenosis detected by coronary CT angiography targeting 350 HU using a CT number-controlling system. We measured 0-to-0 HU distances in the cross-sectional coronary images of 32 patients. We analyzed the ratio of 0-to-0 HU distances in CT images to media-to-media distances in IVUS images (C:I ratio). The area of ≥0 HU for 103 representative points in the remaining 20 patients was compared to the area of the traced external elastic membrane (EEM) in IVUS images.

**Results:**

There was a strong correlation between 0-to-0 HU distance in CT images and media-to-media diameter in IVUS images (r = 0.97, p<0.001). The C:I ratio was 1.1. EEM area was estimated by dividing the area of ≥0 HU by the square of C:I. There was also a strong correlation between the estimated EEM area and the EEM area in IVUS images (r = 0.95, p<0.001).

**Conclusions:**

Media-to-media diameter and EEM area can be estimated by CCTA targeting the optimized intracoronary CT number when blood vessel borders are defined at 0 HU.

## Introduction

The diagnostic accuracy of coronary computed tomography angiography (CCTA) for coronary artery stenosis is now as good as that of invasive coronary angiography [1]. The ability to perform quantitative analysis of coronary arteries with CCTA may alter diagnostic and treatment strategies for coronary artery disease. For example, stent size may be decided on the basis of CT instead of intravascular ultrasound (IVUS) images.

**Figure 1:**
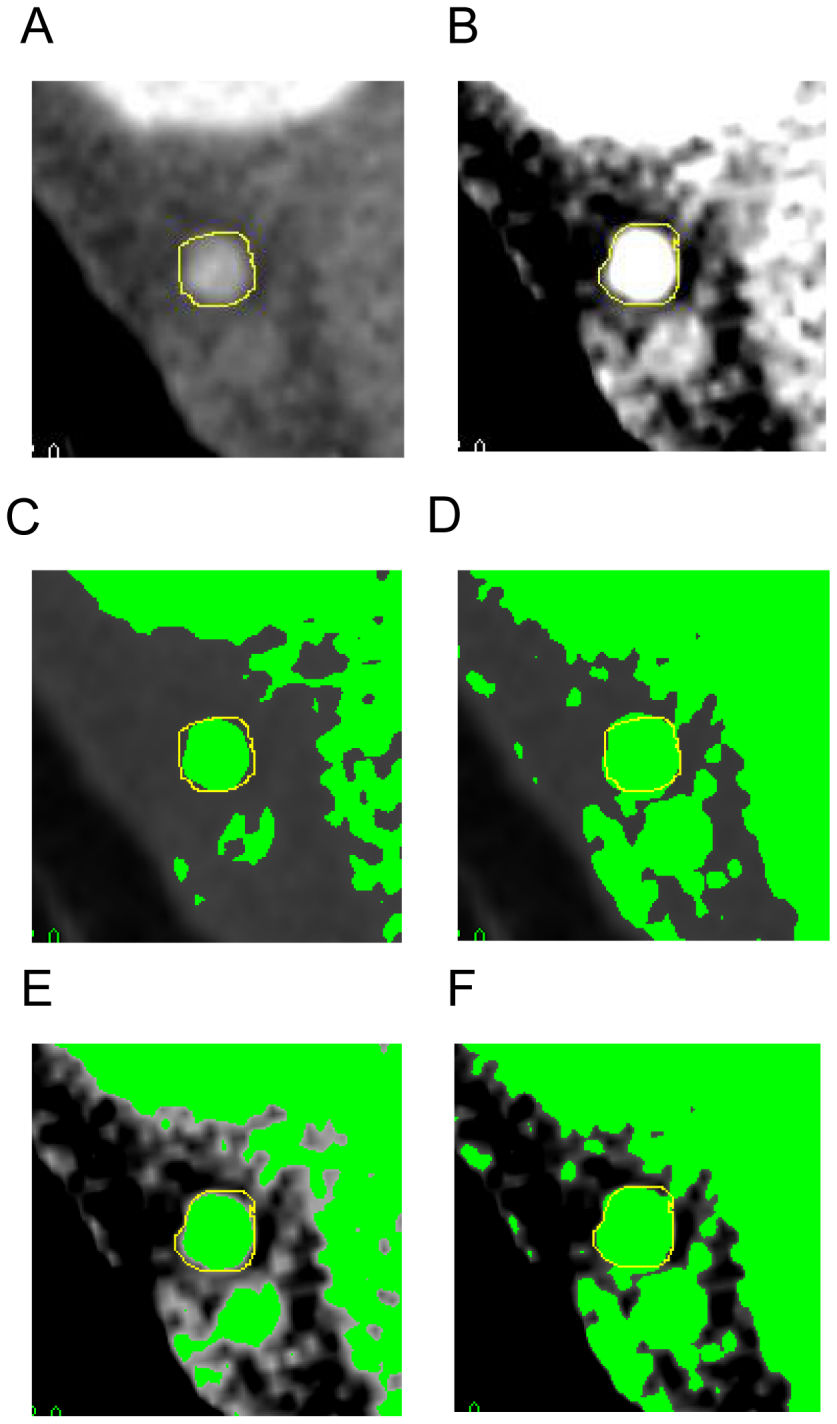
Independent visual determinations of the same cross-sectional image of coronary artery with different window widths/levels.

The quantification of vessel diameters has been attempted [2], [3]. However, the visual determination of vessel borders is plagued by poor reproducibility and inter-observer variability [4]. Marwan et al. [5] reported that the bias in vessel area determinations varied between 65% and 155% for different window widths/levels. Additionally, the visual determination of vessel borders was inaccurate even with the same window widths/levels. Fig. 1A and Fig. 1B are examples of 2 independent visual determinations of the same cross-sectional image of a normal coronary artery with different window widths/levels. Showing the threshold of the image in a different color by Image J software after visual determination of the vessel border, the thresholds of Fig. 1A and Fig. 1B were found to be between −16 HU and 62 HU (Fig. 1C and Fig. 1D) and between −14 HU and 47 HU (Fig. 1E and Fig. 1F), respectively.

Media-to-media distance and external elastic membrane (EEM) area are measurable in cross-sectional IVUS images. However, cross-sectional CT images include 3 vessel layers and extra-adventitial tissue that is not distinguishable. On CT images, there is no vessel boundary point or inflection point in the profile curve [6]. Methods for vessel measurement in commercially available workstations have not been published, and there seems to be no universal method of measuring structures in CT images.

A high intracoronary CT number may cause misdiagnosis of coronary stenosis because of a partial volume effect [7]. Thus, an optimized CT number is required to measure vessels. An intracoronary CT number of 350 Hounsfield units (HU) is desirable to achieve a precise diagnosis of coronary stenosis and plaque [7]. However, the optimized CT number has been difficult to obtain. We established a CT number-controlling system [8] that controls the intracoronary CT number for CCTA.

In our study, CCTA targeted 350 HU, the optimized intracoronary CT number for analyzing coronary stenosis and plaque [8]. We set the optimal border as 0 HU for CT imaging and calculated the ratio of the 0-to-0 HU distance in CT images to the media-to-media distance in IVUS images. The feasibility of the ratio was confirmed by comparing the estimated EEM area obtained by dividing the area of ≥0 HU by the square of the ratio with the EEM area in IVUS images.

## Methods

### Study Sample

We prospectively enrolled 56 patients (age, 64±15 years; range, 42–85 years) with significant coronary stenosis and ischemia who underwent percutaneous coronary intervention (PCI) using IVUS. CCTA indications were consistent with the guidelines of the Society of Cardiovascular Computed Tomography [9]. CCTA was performed 1 day to 4 weeks before the PCI.

Patients were randomly divided into the following 2 groups in a 6∶4 ratio: the diameter comparison group and the area verification group. The diameter comparison group comprised 34 patients for whom we calculated the ratio of 0-to-0 HU distance (in CT images) to media-to-media distance (in IVUS images), defined as the C:I ratio. The area verification group comprised 22 patients, whose data we used to evaluate the feasibility of the C:I ratio for correcting the ≥0 HU area to estimate the EEM area.

This study was approved by the ethics committee of Amagasaki Central Hospital, and all patients provided written informed consent. Clinical exclusion criteria for CCTA were contrast media (CM) allergy, renal insufficiency (glomerular filtration rate <60 mL/min/1.73 mm^2^), pregnancy, congenital heart disease, and coronary artery bypass graft.

Patients were administered an oral beta-blocker (25 mg Atenolol, AstraZeneca, Osaka, Japan) 120 min before CCTA and 0.3 mg of sublingual nitroglycerin 5 min before CCTA. Heart rate was maintained between 55 and 65 bpm through the administration of additional intravenous beta-blocker (2–10 mg Propranolol, AstraZeneca).

### Scanning Protocol

We used a 64-detector CT scanner (Lightspeed VCT; GE Healthcare; Milwaukee, USA) with electrocardiogram (ECG) gating and Advantage Workstation 4.3 software. The scan sequence was as follows: scanogram, scout, test scan, and coronary angiography.

Patients underwent CCTA with a prospective ECG-triggered axial scan (Snapshot Pulse) with the following parameters: slice acquisition, 64×0.625 mm (40-mm volume coverage); 75% of the RR interval with 0–100 ms of padding; rotation time, 350 ms; temporal resolution, 175 ms; tube voltage, 120 kV; and tube current, 450–680 mA. Retrospectively ECG triggered CCTA was selected if difficulties were encountered in maintaining the heart rate at 55–65 bpm or if blurring was detected by the ECG-triggered scanogram. ECG modulation of 40–80% of the RR interval was used. The pitch was approximately 0.2–0.3.

Contrast medium (CM) (350 mg I/mL; Omnipaque 350; Daiichi Pharmaceutical Co, Ltd, Japan) was injected into an antecubital vein at 4 mL/s with a saline chaser using a dual-headed injector system (Dual Shot GX, Nemoto Kyorindo Co, Ltd, Tokyo, Japan) during the timing bolus and CCTA. The amount of CM was determined with a CT number-controlling system, as described elsewhere [7]. CM (5 mL) with 40 mL of a saline chaser was used as a timing bolus for determining the circulation time for the enhanced scan. Sequential scans were obtained every 2 s, from 10 to 40 s after the timing bolus. The region of interest (ROI) was located in the ascending aorta, and a time-density curve (TDC) was determined. The peak time was identified, and the absolute value of the CT number of the ascending aorta at the peak time was measured at the ROI. A scan delay was defined as 2 s after peak enhancement. The determined amount of CM with 40 mL of a saline chaser was administered for CCTA.

The TDC was considered valid if it was a unimodal curve with a measurable peak time and peak CT number. The TDC was invalid if it was flat or a multimodal curve, or if the peak time and peak CT number could not be determined.

### Data Selection

One reader enrolled vessel lesions of different sizes from each patient. The 0-to-0 HU distances on the enrolled CT images were determined by 2 readers who had evaluated the cardiovascular CT images of at least 500 cases to examine inter-observer variability. Curved multiplanar reformation images were used to determine the candidates. Lesions with stents or with a calcified plaque size measuring less than half the diameter of the vessel were excluded owing to the possible presence of a hypershooting artifact. Lesions with blurring artifacts or motion artifacts were excluded. Lesions with a myocardial bridge or those in contact with the myocardium, branches, or veins were excluded because the border of a vessel with the myocardium, branches, or veins showed more than 0 HU. Lesions with 1.5 mm of 0–0 HU distance were excluded.

### The Border-0 Method

**Figure 2:**
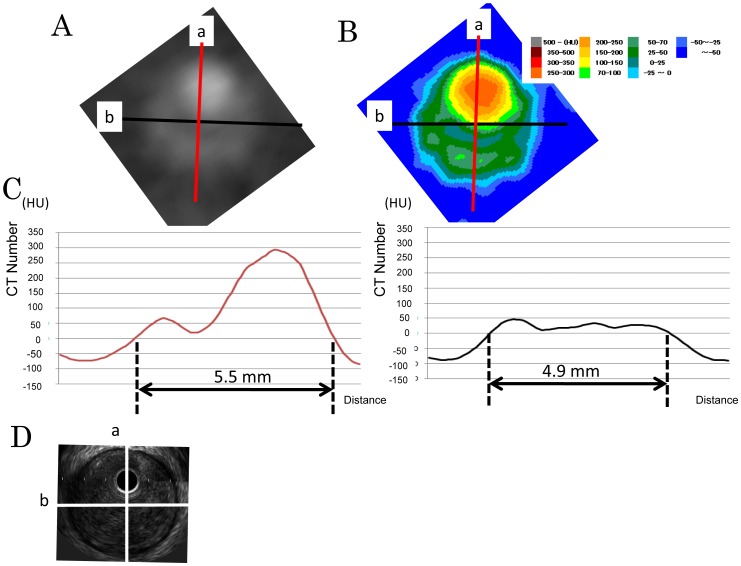
Quantitative analysis of grayscale image (A), Plaque Map (B), Profile curve (C) and IVUS image (D).

Cross-sectional images (256×256 pixels) were constructed using commercial software (Advantage Workstation 4.6, GE Healthcare; Milwaukee, USA). The field of view was magnified between 22 and 30 mm for recognizing the shape of the image, depending on the software. Changing the size of the image does not influence the measurements (data not shown). Because there is no inflection point indicating the border of a vessel on CT images, we established the border-0 method: we set the optimal border as 0 HU and measured the C:I ratio, which is the ratio of the 0-to-0 HU distance in CT images to media-to-media distance in IVUS images. We manually compared cross-sectional images of the long axis and short axis of the vessel (Fig. 2A) with a Plaque Map [10] (Fig. 2B) and analyzed the 0-to-0 HU distance in the long axis and short axis by profile curve of perpendicular straight line a (left) and line b (right) with Image J software 1.43u (US National Institutes of Health) (Fig. 2C). The 0-to-0 HU distance was 5.5×4.9 mm, and the media-to-media diameter in the IVUS image was 5.0×4.4 mm. (Fig. 2D).

We performed IVUS with a 40-MHz, 2.9-Fr IVUS catheter (Atlantis, Boston Scientific; Natick, MA, USA) with 0.5 mm/s automatic pullback. Media-to-media distances were analyzed by 2 readers with at least 5 years of IVUS experience to examine inter-observer variability.

Anatomic cross-correlation between MDCT and IVUS images was achieved by using the nearest bifurcation points as reference markers and by taking plaque shape into account (Fig. 2D). We rotated either the CT image or the corresponding IVUS image until the location of the plaque components matched. A line was drawn for the 0-to-0 HU distance such that it did not penetrate the calcification and surrounding area, because the quite high CT number of the calcification enhances the CT number of the surrounding area. We excluded CT images containing calcifications of more than 90° or motion artifacts. IVUS images with nonuniform rotational distortion or catheter artifacts and those with calcification-generated shadows were also excluded. Media-to-media distances on IVUS images were traced manually on IVUS images displayed with Image J software. Correspondences between CT and IVUS images were initially determined by 1 reader. If anatomical discordance was present between them following evaluation by the second reader, the readers continued to individually evaluate the images until consensus was reached.

We calculated the ratio of the 0-to-0 HU distance in cross-sectional vessel images (Fig. 2C) to the media-to-media diameter in IVUS images (Fig. 2D).

### The Feasibility of the C:I Ratio for Estimating EEM Area

If the ratio of the 0-to-0 HU distance on CT image to the media-to-media distance on IVUS image is constant, the EEM area in IVUS images can be estimated by the ≥0 HU area in CT images by correcting with the C:I ratio. The estimated EEM was defined as the ≥0 HU area on CT image divided by the square of the C:I ratio. The estimated EEM areas determined by CT were compared to the EEM areas determined by IVUS in the area verification group.

**Figure 3:**
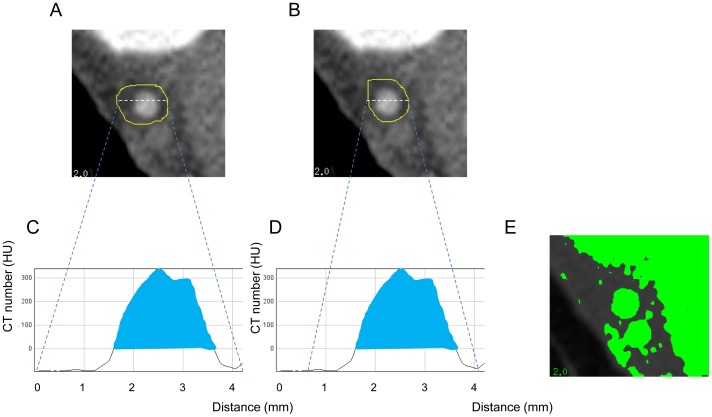
Different traces (A, B), the region (C, D) and the image (E) of of the ≥0 HU.

The region that included the vessel was manually traced wider than the outside of the vessel, as shown in Fig. 3. The analysis of the ≥0 HU area for estimated external elastic membrane by the Border-0 method. The region including the vessel was manually traced wider than the outside of the vessel. (A, B) Two independent manual traces (yellow lines) wider than the outside of the vessel. (C, D) Profile curves of white dotted lines in Fig. 3A, 3B. The ≥0 HU areas (shown in blue) are the same. The ≥0 HU areas of Fig. 3A, 3B are 27.0 mm^2^ and 27.3 mm^2^, respectively. (E) The threshold of 0 HU of Fig. 3A was shown in a different color. If the trace does not include stained myocardium, the ≥0 HU areas of any manual traces may have good reproducibility. A custom macro in Excel 2007 for Windows 7 calculated the ≥0 HU area in the selected area exported as text data from Image J.

### Statistical Analysis

Statistical analysis was performed using SPSS 17.0 (SPSS Inc.; Chicago, Illinois). Data are expressed as means ± SD. We used linear regression analysis and Bland and Altman’s analysis of agreement method to assess the association between 0-to-0 HU distance in cross-sectional images and media-to-media distance in IVUS images. We used the same methods to assess the association between estimated EEM area in CT images and EEM area in IVUS images.

## Results

### Patient Characteristics

Table 1 summarizes patient characteristics and CCTA conditions. Two patients in the diameter comparison group and 2 from the area verification group had invalid TDCs and were excluded from the analysis, because our system cannot control the intracoronary CT number in such cases. The amount of contrast media averaged 36±5 mL in the diameter comparison group and 34±6 mL in the area verification group. The average intracoronary CT number was 349±38 HU in the diameter comparison group and 352±36 HU in the area verification group.

**Table 1:** Patient Characteristics and Coronary Computed Tomography Angiography Conditions.

	Diameter comparing group	Area verification group
N	34	22
Gender (M%)	7 (21%)	4 (18%)
Age	68±14	66±15
Hypertension	16 (47%)	12 (54%)
Hyperlipidemia	26 (76%)	14 (63%)
Diabetes	2 (6%)	2 (9%)
Smokers	14 (41%)	8 (36%)
Stable angina pectoris	21 (62%)	14 (64%)
Unstable angina	13 (38%)	8 (36%)
Valid time-density curve at timing bolus	32 (94%)	20 (91%)
Amount of contrast media (mean ± SD, mL)	36±5	34±6
Intracoronary CT number (mean ± SD, HU)	349±38	352±36

SD: standard deviation; HU: Hounsfield unit.

### Correlation between 0-to-0 HU Distance and Media-to-media (IVUS) Distance

**Figure 4:**
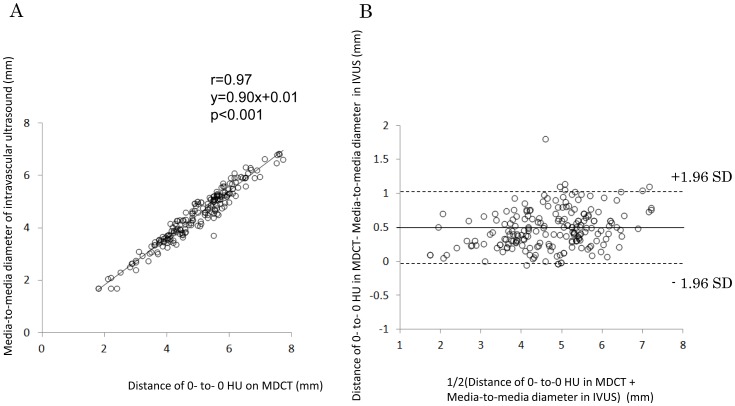
The correlation between 0-to-0 HU distance on MDCT (x) and media-to-media (IVUS) distance (y).

We enrolled 238 slices from 32 patients in the diameter comparison group. Thirty-three slices were excluded. We examined 205 slices (86%) and found a strong correlation between 0-to-0 HU distance (x) and media-to-media (IVUS) distance (y) (Fig. 4A) (y = 0.90x+0.01, r = 0.97, p<0.001). A Bland–Altman plot is shown in Fig. 4B. Mean difference was −0.48, limits of agreement was −0.08 to 1.04. Inter-observer variability was 0.88±2.45% for 0-to-0 HU distance and 0.75±3.00% for media-to-media distance. The 0-to-0 HU distance was 10±5.3% larger than the media-to-media distance. Thus, the media-to-media distance in IVUS images can be estimated by dividing the 0-to-0 HU distance in MDCT images by 1.1, and EEM area in IVUS can be estimated by dividing the ≥0 HU area in CT images by the square of 1.1.

### Correlation between Estimated EEM Area and EEM Area in IVUS Images

**Figure 5:**
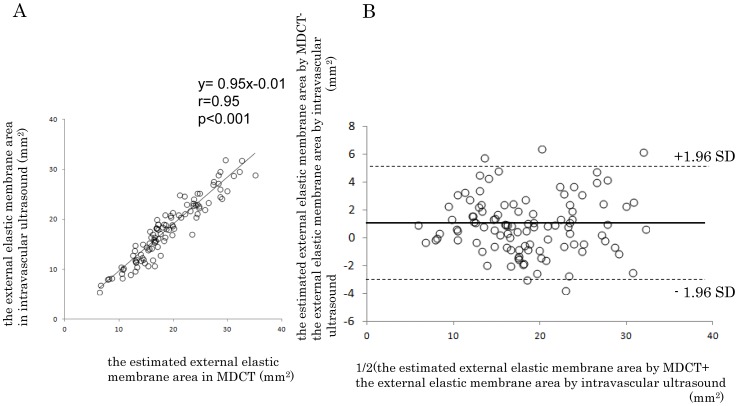
The correlation between the estimated and the measured EEM area (A), and Bland-Altman plot (B).

We examined 126 slices from 20 patients from the area verification group. Twenty-three slices were excluded. We examined 103 slices (82%) to assess the correlation between estimated EEM area in CT images and EEM area in IVUS images (Fig. 5A). The C:I ratio was 1.1, and the square of the ratio was 1.21. There was a strong correlation between the estimated EEM area (x) and the EEM area in IVUS images (y) (y = 0.95x+0.19). A Bland–Altman plot is shown in Fig. 5B. Mean difference was 1.00, limits of agreement was −3.01 to 5.01. The difference between the estimated EEM area in CT images and the EEM area in IVUS images was 9.0±14%.

## Discussion

The ability to quantify vessels with MDCT is valuable, for example, for deciding on stent size and evaluating the change of positive or negative remodeling. Three problems seem to prevent precise measurement with MDCT images: variability of quantitative measurements according to the cardiac cycle, variable intracoronary CT number, and lack of standard methods for measurement with CT images.

The diastolic cardiac cycle was used for CT images, but every cardiac cycle was used for IVUS images in our study. The difference does not seem to be significant because only a 2.0% to 5.7% difference in luminal area was observed between the systolic and diastolic phases in diseased coronary artery segments, and the difference was not statistically significant in other studies [11], [12]. Moreover, variability of quantitative measurements according to the cardiac cycle is not considered to determine stent size in the clinical setting.

CT number and diameter were thought to be unreliable because CT numbers and spatial resolutions vary [6]. The CT number of the coronary lumen is influenced the most. A high intracoronary CT number may amplify the plaque CT number [13]. Recent reports have proposed that the amount of CM should be adapted to body surface area [14] or body mass index [15] rather than to body weight for better prediction. However, the coronary CT number for a specific amount of CM differed by more than 200 HU with these methods. The SD of a CT number targeting 350 HU by the CT number-controlling system decreased; in 2 groups, it was only 38 HU and 36 HU, respectively. Using the CT number-controlling system, the difference of intracoronary CT numbers between the second and first CCTAs was the smallest when compared to CCTAs using the same contrast volumes or constant volumes per body weight [16].

Owing to the partial volume effect and extra-adventitial tissue, determining the border of the coronary artery from CT images has been difficult. An additional disadvantage of IVUS is that the borders of media and adventitia are measurable, but the actual vessel diameter is not because adventitia and adventitial tissues are indistinguishable [17]. Therefore, we set 0 HU as the marker for borders on CT images and calculated the ratio of 0-to-0 HU distance to media-to-media distance. The ≥0 HU areas of any manual traces wider than the outside of the vessel may have good reproducibility if the trace does not include stained myocardium.

The tentative available border might be −10 HU or −50 HU instead of 0 HU. The C:I ratio might be greater than 1.1. If the border is defined at 50 HU or 100 HU for obtaining a C:I ratio close to 1, most of the area of lipid-rich plaque might be truncated. The border 0 HU has the advantage of accuracy because 0 HU is one of the standards of a CT image. Measurements that are manually traced on CT images are fundamentally inaccurate because the CT number of the borders is not fixed, and such measurements are inflated compared to those made with IVUS. It is difficult to set the fixed border between the vessel and plaques, which shows varied CT numbers of approximately 60 to 80 HU (Fig. 1). Therefore, measuring plaque area has been difficult so far.

Using a ratio of 1.1 and targeting an intracoronary CT number of 350 HU, we found a strong correlation between estimated EEM area and EEM area measured with IVUS, which differed by 9.0±14%. The difference, for instance, between a vessel diameter of 0.09 mm and a vessel diameter of 3 mm is not clinically significant. The slight computational error may be due to the conditional difference in vessel dilatation between CT and IVUS.

### Study Limitations

We excluded invalid TDCs at the timing bolus. In such cases, our CT number-controlling system cannot predict the intracoronary CT number because of a lack of data. We quantified plaque in a small subset of the patients because only patients with both significant stenosis detected by MDCT and evidence of ischemia had been enrolled. More studies are needed in other subtypes of patients in order to identify the optimal intracoronary CT number.

We had few data of less than 2 mm in diameter for 2 reasons. One is that small coronary arteries were excluded because small coronary arteries are often in contact with the myocardium. The other is that the measurement of a vessel less than 2 mm in diameter has little clinical significance because diseased lesions with 2 mm of diameter are treated by medication instead of percutaneous coronary intervention. Measurement by CT and IVUS was performed to avoid the calcification and surrounding area. The calcified area with shadowing is also immeasurable by CT and IVUS. The number of measurable lesions may depend on the extent of calcium deposition or the number of stents. In clinical settings, that the location of measurement is limited may be inevitable with both IVUS and CT.

## Conclusions

Media-to-media distance and EEM area in IVUS images can be estimated by CCTA targeting the optimized intracoronary CT number when blood vessel boundaries are defined at 0 HU.
